# A Mapping Sentence for Understanding the Genre of Abstract Art Using Philosophical/Qualitative Facet Theory

**DOI:** 10.3389/fpsyg.2017.01731

**Published:** 2017-10-12

**Authors:** Paul M. W. Hackett

**Affiliations:** ^1^School of Communication, Emerson College, Boston, MA, United States; ^2^Department of Psychology, University of Gloucestershire, Cheltenham, United Kingdom

**Keywords:** perception of art, experience of art, facet theory, mapping sentence, mereology, ontology, aesthetics, art

Whether we are philosophers or are members of the general public, during the course of our daily activities we sub-divide our phenomenological world and form categorial accounts of these experiences. The development of categorial ontologies, or sub-divisions of the most basic levels of our existence, has long been used to enable a clearer understanding of a specific domain of interest. Similarly, ontological scholarship has a long and distinguished history which continues to this day (see for example: Aristotle and Ackrill, 1975; Simons, 1987; Harte, 2002; Sider, 2005; Lowe, 2007; Chisholm, 2010; Poli and Seibt, 2010). Coffey (2016) provides an overall contemporary review of the use of ontologies by philosophers.

In Hackett (2016a) I claimed that facet theory research (Canter, 1985) may be considered a form of meta-ontological enquiry and analysis due to it incorporating the mapping sentence as its guiding structure: The mapping sentence is a template within which the researcher overtly states the major sub-divisions of an area of research interest. I further asserted that facet theory embodied notions of a meta-mereology as the mapping sentence also makes much of the way in which the sub-components of a research domain (the basic ontological units which are called facets) are broken-down into mutually exclusive “elements.” More precisely, the inter-relationships between facets and elements are stated linguistically in the mapping sentence. Earlier, in Hackett (2014) I have already argued for the utility in developing a qualitative or philosophical approach to facet theory^1^ and suggested that this is best thought of as a meta-mereology.

The mapping sentence links together the pertinent components of a research domain in such a way that the variables (facets) and sub-components of the variables (elements) are combined using every day prose so as to suggest the inter-relationship between facets and elements in the context of a specific research undertaking. Hackett (2016a,b, 2017) has used a qualitative or philosophical facet theory approach to facilitate an account of perceiving different forms of abstract art. Through amalgamating existing theory within this research domain along with empirical observations, the mapping sentence has the potential to extend psychological and philosophical knowledge and understanding of how abstract forms of modern and contemporary fine art are perceived and experienced. In the above-mentioned articles I addressed abstract art that was either two-dimensional (Hackett, 2016b) or three-dimensional (Hackett, 2017). In the latter of these publications I suggested that the findings from these two branches of research might be brought together to suggest a way to investigate abstract art as a united genre. Below, I address this claim.

Paul Crowther is philosopher who has developed ontological accounts in his research. Of specific interest to the claims I make in this paper, is his book of 2007 Defining Art, Creating the Canon: Artistic Value in an Era of Doubt. Crowther (2007) first puts forward and then justifies the notion that art can be thought of in terms of its ontological components. He proposed an eight part ontology that is made-up of the following categorical characteristics: resemblances—joining, connecting, uniting in an advantageous or rewarding way, colors, shapes and textures so that these resemble certain visual configurations and shapes (e.g., images in cloud patterns); gestural associations—symbolic relationships, connections with visual manifestations that arouse states of mind (e.g., violent shapes, depressing colors); revelations—aspects of articles, objects, associations, tiny small surface features, internal configurations, fleeting atmospheric effects, unusual perspectives, and other events that are not usually visible; novel environments—articles, objects, associations and other events, located in perceptual and physical environments that they are not usual found in; neoteric configurations—bringing about of a visual array, positioning or arrangement through destruction, deconstruction, reduction, reconstruction or in some way altering familiar events; visual suggestions—previous, future or counterfactual events, items or states of affairs arising from visual lines, colors, shapes, symbols or suggestions; spatiality/structure—visual spatial impression and configuration of attributes, such as: color, shape, volume, mass, texture, density, geometric structure, alterations in positions, either alone or in combination; fantasy—a state of unreality or hallucinatory circumstances and appearances.

However, Crowther's comprehensive ontology does not suggest a combinatorial rational for its elements. In this essay I consider the validity of Crowther's characteristics to all two- and three-dimensional abstract art and offer a categorial ontology that considers mereological aspects of these basic units of art experience. However, I first consider my earlier work that exclusively looked at two- and three- dimensional abstract art. In Hackett (2016b) I report research into the two-dimensional format of abstract and found that six of Crowther's eight characteristics legitimately structured perceptions of this art genre. The six legitimate Crowther characteristics were: resemblances; gestural associations; revelations; novel environments; visually suggestions and spatiality/structure. The two characteristics of neoteric configurations, and fantasy did not appear to play an important role in structuring understanding. I later considered Crowther's characteristics in terms of three-dimensional abstract art. Again, not all of the eight characteristics played an important role in structuring understanding of these art works. In this instance the pertinent characteristics were: resemblances; novel environments; visually suggestions; and spatiality/structure features. The feature of fantasy appeared to play a minor role and along with the characteristics of gestural associations, revelations and neoteric configurations were of little importance in structuring understanding. Thus, I believe that it is possible to usefully combine my findings in regard to two- and three-dimensional abstract art. In doing this it is possible to facilitate a depiction and offer understanding of abstract art as an overall genre of art.

Thus, I propose that Crowther's characteristics may be reduced in number as through using smallestspace analysis not all of these were found to structure my appraisals. Consequently, Crowther's characteristics may be reduced in number to those that partitioned both two- and three-dimensional abstract art. These were: resemblances; novel environments; visually suggestions; spatiality/structure. In Figure 1, a mereological arrangement of the combination of these four facets and their respective elements of experience is proposed in the format of a mapping sentence.

**Figure 1:**
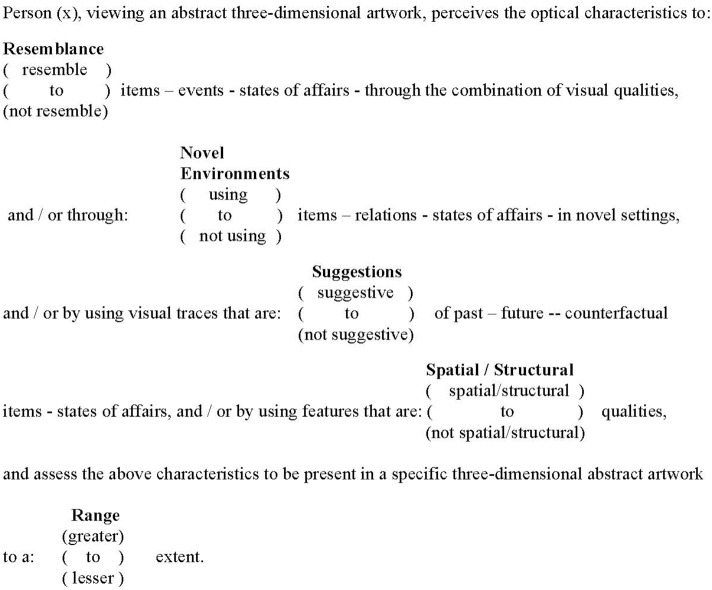
Mapping sentence for understanding the experience of abstract art.

By using the above mapping sentence, it is possible for an individual painting, drawing, sculpture, installation, piece of land art, etc., to be depicted in terms of its structuples (profile of facets and its elements). Furthermore, if the facets and elements that have been incorporated into the mapping sentence are both valid and comprehensively address the domain of abstract art this account will yield, through structuple combinations, a total definition of the phenomena of the abstract art object.

It is important to note that the combination of pertinent characteristics of how we perceive and understand both two- and three-dimensional abstract art may suggest an overall framework for abstract art perception. However, this statement requires investigation as the identification of characteristics that were employed for understanding the two forms of abstract art when viewing either two- or three- dimensional abstract art works may not combine in a meaningful manner when a person observes two- and threedimensional abstract together. The question as to whether the combination of the findings to study the combined genre of abstract art experience is an area of ongoing research. This caveat notwithstanding, it seems that the combination of the pertinent characteristics for perceiving two- and three-dimensional abstract art, in a mapping sentence format, at the very least provides a framework that will facilitate further enquiry.

I justify my optimism by noting how the mapping sentence is a tool that I have used to investigate fine art within several different and specific contexts including art objects (Hackett, 2013) and art education (Schwarzenbach and Hackett, 2015) which has enabled me to presented multiple mapping sentences for these different aspects of fine art. From these mapping sentences, I believe it is reasonable to state that the mapping sentence is a framework that may facilitate research that clearly addresses a variety of contextualized art experience. However, this research is in its infancy and is subject to ongoing study and further consideration.

My work into the area of facet theory as a qualitative and philosophical approach (Hackett, 2013, 2014, 2016a,b, 2017)^2^ is also supported by my research into abstract art. These publications extend the facet theory literature and support the use of the mapping sentences as meta-ontological and meta-mereological structure within which reliable, valid, consistent and cumulative research may be undertaken and knowledge developed.

I have written this opinion article in an attempt to encourage the investigation of the highly intricate research and experiential domain of visual perception when this is applied specifically to our understanding of abstract art. I have suggested that a faceted understanding of this categorial experience instantiates the multifarious nature of art perceptual experiences. The findings of my research into abstract art require the question to be asked as to whether non-abstract art may be understood using the same mapping sentence? It is obvious that representational art embodies notions of likeness. However, a facet that reflected the similarity of an ort object to the event or thing it is representing could be incorporated into the mapping sentence. Even when experiencing the most representational of art objects, perhaps a photographic portrait, this representation will involve other associations that result from the art object being in experiential dialog with the viewer and which are suggested in the four facets in the Mapping Sentence for Understanding the Experience of Abstract Art (Figure 1). Consequently, the facets contained in this mapping sentence may provide a template that can be adapted and used to investigate non-abstract art. What I am claiming is that the mapping sentence investigated within a qualitative and philosophical framework provide a template for understanding the complexities of the perception and understanding of art.

## Author contributions

The author confirms being the sole contributor of this work and approved it for publication.

### Conflict of interest statement

The author declares that the research was conducted in the absence of any commercial or financial relationships that could be construed as a potential conflict of interest.

## Footnotes

^1^On such an approach the data that arises will likely come from individual reflection (as is the case of the analyses reported in this essay, or will come from small sample sizes). In the research reported in this essay, smallestspace analysis (SSA) was used to analyse the data in the form of a case study.

^2^In my current research I have been using a philosophical/qualitative approach to facet theory research in combination with traditional quantitative facet theory procedures in the investigation of avian cognition. The initial results of this approach appear to be providing insight into avian behavior.
